# Consequence of Menin Deficiency in Mouse Adipocytes Derived by In Vitro Differentiation

**DOI:** 10.1155/2015/149826

**Published:** 2015-07-02

**Authors:** Vaishali I. Parekh, Sita D. Modali, Shruti S. Desai, Sunita K. Agarwal

**Affiliations:** Metabolic Diseases Branch, National Institute of Diabetes and Digestive and Kidney Diseases, National Institutes of Health, Bethesda, MD 20892, USA

## Abstract

Lipoma in patients with the multiple endocrine neoplasia type 1 (MEN1) syndrome is a type of benign fat-cell tumor that has biallelic inactivation of *MEN1* that encodes menin and could serve as a model to investigate normal and pathologic fat-cell (adipocyte) proliferation and function. The role of menin and its target genes in adipocytes is not known. We used in vitro differentiation to derive matched normal and menin-deficient adipocytes from wild type (WT) and menin-null (Men1-KO) mouse embryonic stem cells (mESCs), respectively, or 3T3-L1 cells without or with menin knockdown to investigate cell size, lipid content, and gene expression changes. Adipocytes derived from Men1-KO mESCs or after menin knockdown in 3T3-L1 cells showed a 1.5–1.7-fold increase in fat-cell size. Global gene expression analysis of mESC-derived adipocytes showed that lack of menin downregulated the expression of many differentially methylated genes including the tumor suppressor long noncoding RNA Meg3 but upregulated gene expression from the prolactin gene family locus. Our results show that menin deficiency leads to fat-cell hypertrophy and provide model systems that could be used to study the regulation of fat-cell size.

## 1. Introduction

Patients with the multiple endocrine neoplasia type 1 (MEN1) syndrome carry a heterozygous germline inactivating mutation in* MEN1* that predisposes to tumors of multiple endocrine and nonendocrine tissues (OMIM ID 131100 and 613733) [1–3]. Lipoma in patients with MEN1 occurs as an overgrowth of fat tissue, frequently multiple, and usually in the subcutaneous area of the back, neck, shoulder, arm, thigh, or abdomen [4, 5]. The MEN1-associated lipoma is a type of benign fat-cell tumor occurring in about 30% of MEN1 patients and similar to the other MEN1-associated endocrine and nonendocrine tumors has biallelic inactivation of* MEN1* (1st hit in the germline, 2nd hit tissue-specific) [5–9]. Lipoma tissue samples are rarely available for analysis because the tumor is harmless and removed only for cosmetic reasons by surgery or liposuction, and the tumor does not recur after removal. In the mouse models of* Men1* loss generated in different laboratories, the endocrine tumors of MEN1 are frequently observed [10, 11]. However, lipoma was observed only in one mouse [12]. Therefore, there is a dearth of molecular studies exploring the pathogenesis of MEN1-associated lipoma.

The* MEN1* encoded menin is a widely expressed, predominantly nuclear protein involved in transcriptional regulation and other vital cellular functions [13, 14]. Comparing gene expression changes in normal and menin-deficient fat-cells (adipocytes) could provide insights into the pathogenesis of MEN1-associated lipomas; at the same time such studies could also provide insights about normal adipocyte growth and function. No such studies have been performed perhaps due to the limitation of procuring normal adipocytes that can serve as a matched control for the abnormal adipocytes in the lipoma. Specific cell types produced by in vitro differentiation of embryonic stem cells (ESCs) or induced pluripotent stem cells (iPSCs) that are normal or mutant for a disease-causing gene can serve as useful models to study molecular processes that are altered in disease [15, 16]. This is highly desirable for affected cell types that are not easily available, are difficult to isolate, and are not abundant or that lie among other normal cell types. Menin-null (Men1-KO) mouse ESCs (mESCs) can differentiate in vitro into pancreatic islet-like endocrine cells (PILECs); however, they are defective in hematopoietic differentiation [17, 18]. It is not known whether Men1-KO mESCs can differentiate into adipocytes.

Gene expression analyses of wild type (WT) and Men1-KO mESCs, and mESC-derived WT and Men1-KO PILECs have shown a role of the menin-dependent histone H3 lysine-4 trimethyl mark (H3K4me3) in the regulation of genes clustered at the* Meg3* locus and the four* Hox* gene clusters [18]. Most gene clusters are highly conserved, such as the four* Hox* gene clusters [19]. However, the prolactin family gene cluster is present in rodents and not in humans [20]. In humans, the prolactin gene resides alone in a gene poor region of the genome on chromosome 6. In rodents, gene duplication has generated a large prolactin gene family of 26 genes near the prolactin gene (mouse chromosome 13) with varying expression and functions in pregnancy and lactation [21, 22].

In this study, we show that adipocytes could be generated by in vitro differentiation of Men1-KO mESCs and upon menin knockdown from 3T3-L1 preadipocyte cells. These menin-deficient adipocytes and their corresponding normal adipocytes were analyzed for cell size, lipid content, and gene expression changes. Menin-deficient adipocytes from both cell types showed increased cell size with a modest increase in lipid content; and adipocytes derived from Men1-KO mESCs showed reduced expression of a few differentially methylated genes including the long noncoding RNA Meg3 and increased expression of prolactin family genes. These cells could enable further investigations by genomic and proteomic approaches to understand physiological and pathological states of adipocytes.

## 2. Materials and Methods

### 2.1. Primers

All the primers used in this study are provided in Supplementary Table  1 in Supplementary Material available online at http://dx.doi.org/10.1155/2015/149826.

### 2.2. Differentiation of Mouse Embryonic Stem Cells (mESCs) into Adipocytes

Adipogenesis was performed by using the reagents and protocol from a mESC adipogenesis kit (Millipore). Wild type TC-1 and Men1-KO-3.2N mESCs [17] were cultured on a feeder layer of primary mouse embryonic fibroblasts (MEFs) (ATCC) in ESC maintenance medium containing leukemia inhibitory factor (LIF). The mESCs were processed to obtain feeder-free cells by culturing on tissue culture treated dishes few times for an hour each to get rid of the attached feeder layer cells, and the unattached feeder-free mESCs were subsequently cultured on gelatin-coated dishes in ESC maintenance medium containing LIF. Cells were detached with accutase and plated in embryoid body (EB) formation medium (EB medium) (ESC maintenance medium without LIF) in nonadherent dishes for 2 days and then in medium containing inducer-A solution (all-trans-retinoic acid) for 3 days with a medium change every day. On day 5, the retinoic acid induced EBs were plated in gelatin-coated 24-well plates in adipocyte differentiation medium (EB medium + 3,3′,5-triiodo-L-thyronine (T3) + insulin). The adipocyte differentiation medium was changed every 2 days for a total of 21 days to allow adipocyte formation.

### 2.3. Transfection and Differentiation of 3T3-L1 Cells into Adipocytes

3T3-L1 cells (ATCC) were cultured in high glucose Dulbecco's Modified Eagle's Medium (DMEM) supplemented with 10% Fetal Bovine Serum (FBS) and 1X antibiotic/antimycotic (3T3-L1 cell propagation medium). For menin knockdown, 1 × 10^6^ cells were electroporated with 10 *μ*g of control shRNA plasmid or Men1-shRNA plasmid [23] by using the Nepa Gene Super Electroporator NEPA21 Type II (Bulldog-Bio) (Porting Pulse: volts (V) = 175, length milliseconds (ms) = 5, interval (ms) = 50, number = 2, and decay rate (%) = 10; transfer pulse: *V* = 20, length (ms) = 50, interval (ms) = 50, number = 5, decay rate (%) = 40, and polarity = +/−). Electroporated cells were plated in 3T3-L1 cell propagation medium overnight and induced to differentiate into adipocytes using an established protocol [24]. On the next day medium was changed to adipocyte differentiation medium (3T3-L1 cell propagation medium with isobutylmethylxanthine, dexamethasone, and insulin) and incubated for 2 days. Thereafter the medium was changed to maintenance medium (3T3-L1 cell propagation medium with insulin) for the next 4 days with a change of medium every 2 days to allow adipocyte formation.

### 2.4. Oil Red O Staining and Lipid Content

Adipocytes obtained after 21 days of mESC differentiation were processed for Oil Red O staining by using the reagents provided in the mESC adipogenesis kit (Millipore). Oil Red O specifically stains lipids such as triglycerides and cholesteryl oleate [25]. For adipocytes derived from 3T3-L1 cells, Oil Red O (Sigma) reconstituted in isopropanol was diluted 3 : 2 with distilled water (dH_2_O). Cells in the culture dish were rinsed with 1X Dulbecco's Phosphate-Buffered Saline (DPBS) and fixed with 4% paraformaldehyde for 20 minutes and then rinsed with 1X DPBS. Oil Red O staining solution was added and the cells were allowed to stain for 20 minutes. Cells were rinsed 4 times with 1X DPBS. To determine lipid accumulation in the adipocytes, after aspirating the last 1X DPBS rinse, 0.25 mL dye extraction solution was added to each well to extract the Oil Red O dye from the stained lipids. The plate was allowed to shake on an orbital shaker for 30 minutes and the extracted dye was transferred to a 96-well plate, and optical density (OD) was measured at 520 nm in a plate reader (Molecular Devices).

### 2.5. Microscopy and Cell Size Measurement

Images of Oil Red O stained cells were captured by bright-field microscopy on the Axiovert 40 CFL (Zeiss) or DM5000B (Leica Microsystems) at 400x magnification. The size of Oil Red O stained cells was determined from cells contained in 3–5 microscopy fields per experiment. As an index of cell size, the cell diameter was measured from microscopy images. Microscopic morphological images of the adipocytes were printed, circles were drawn at the periphery of the individual cells around the circular shape formed by the orange lipid droplets accumulated inside the cell, and the diameter of the circles was noted using a ruler [26–28]. The diameter mean and SD were plotted for each experiment.

### 2.6. RNA Isolation, Conventional Reverse-Transcriptase PCR (RT-PCR), Microarray, and Real-Time Quantitative PCR (QPCR)

Total RNA was isolated by using the RNeasy mini kit (Qiagen) and DNase treated by using the Turbo DNA-free kit (Ambion/Life Technologies).

Expression of adipogenesis marker genes (*Adiponectin*,* Pgc1α*, and* Pparγ*),* Men1*, and an internal control gene (*Gapdh*) was determined by conventional RT-PCR using RNA isolated from cells before and after differentiation. OligodT-primed first strand cDNA was used for PCR using the GoTaq Green Master Mix (Promega). Equal amount of PCR products was analyzed by agarose gel electrophoresis.

RNA from WT and Men1-KO mESC-derived adipocytes was analyzed for global gene expression changes at the NIDDK genomics core facility (Affymetrix microarray platform). RNA samples (*n* = 3 of each) were labeled and hybridized to Genechip mouse genome 430 arrays. Microarray data were normalized and analyzed using Affymetrix Genechip software, Microarray analysis Suite 5.0 (Affymetrix). For the validation and further analysis of the target genes identified by microarray, OligodT-primed first strand cDNA was used for QPCR using the Brilliant II SYBR Green QPCR Kit (Agilent Technologies) and Mx3000P PCR machine (Stratagene). To calculate fold change, 2^−ΔΔCt^ values were calculated from the raw threshold values generated by the Mx3000P software. Gapdh was used as the internal control.

### 2.7. Western Blot Analysis

Whole cell extracts (WCE) were prepared in cell culture lysis reagent (Promega), and protein quantitation was performed with the DC protein assay kit (Bio-Rad). Equal amounts of protein were separated on 10% Tris-Glycine gels, transferred to nitrocellulose membrane, and probed with appropriate dilution of primary and secondary antibodies, and the blots were developed using enhanced chemiluminescence (ECL) (Millipore). Antibodies used were rabbit antimenin (Bethyl, A300-105A) and mouse antitubulin (Calbiochem, CP06).

### 2.8. Statistics

Data from at least 3 independent experiments or measurements were plotted as mean and standard deviation (SD). Differences between groups were compared by Student's *t*-tests. *p* < 0.05 was considered significant.

## 3. Results

### 3.1. Men1-KO mESCs Can Undergo In Vitro Differentiation into Adipocytes and Show Increased Adipocyte Cell Size

**Figure 1:**
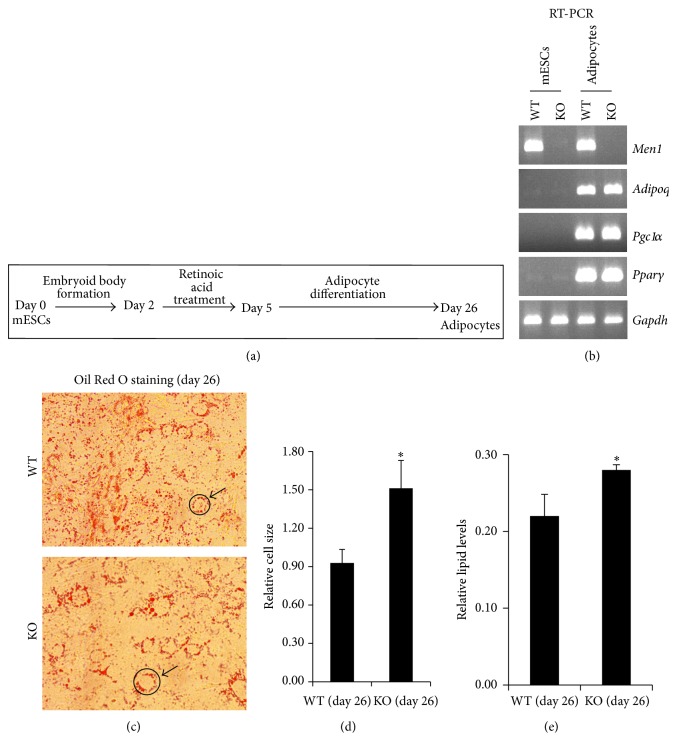
Menin-null (Men1-KO) mouse embryonic stem cells (mESCs) can undergo in vitro differentiation into adipocytes and show increased adipocyte cell size. (a) In vitro adipogenesis. Feeder-free mESCs (WT or Men1-KO) were allowed to form embryoid bodies (EBs) in medium without LIF for 2 days, followed by retinoic acid treatment for 3 days. On day 5, retinoic acid induced EBs were cultured in gelatin-coated plates with adipocyte differentiation medium for 21 days to obtain adipocytes, and the medium was replaced every 2 days. (b) Expression of adipocyte marker genes. Conventional RT-PCR followed by agarose gel electrophoresis of the adipocyte marker genes (*Adiponectin*,* Pgc1α*, and* Pparγ*),* Men1*, and an internal control gene (*Gapdh*) using RNA samples before and after in vitro differentiation of WT or Men1-KO (KO) mESCs into adipocytes. (c) Oil Red O staining. Bright-field microscopy images of WT and Men1-KO mESCs stained for lipids/triglycerides after adipogenesis by Oil Red O staining show round adipocyte cells with lipid droplets (orange colored spots). Magnification = 400x. (d) Relative cell size. After adipogenesis, Oil Red O staining for lipids/triglycerides was performed, and images were captured by bright-field microscopy shown in (c). Cell diameter was measured as an index of cell size from 3 to 5 microscopy fields using the outline formed by the circle of orange lipid droplets accumulated inside the round adipocytes (an example marked with a black circle and arrow is shown in (c)). Diameter mean and SD are shown (^*∗*^ *p* < 0.05). Note: the units on the *y*-axis do not reflect the actual diameter of the cells but they reflect the measurement of the cell diameter from images captured at 400x magnification. (e) Relative lipid content. After adipogenesis, Oil Red O staining for lipids/triglycerides was performed, dye was extracted, and the OD was measured at 520 nm. Relative lipid level (OD at 520 nm) is shown for adipocytes. Error bar = SD. ^*∗*^ *p* < 0.05.

To determine the ability of Men1-KO mESCs to differentiate into adipocytes, in vitro differentiation of wild type (WT) and Men1-KO mESCs was performed using an established protocol (Figure 1(a)) [29]. Both cell types were equally capable of differentiating into adipocytes with similar expression of adipocyte-specific marker genes (*Adiponectin*,* Pgc1α*, and* Pparγ*) and similar level of Oil Red O staining in lipid droplets (Figures 1(b) and 1(c)). Adipocytes derived from Men1-KO mESCs were 1.5- to 1.7-fold larger than WT adipocytes accompanied with a slight increase in lipid content (Figures 1(d) and 1(e)). Thus, in vitro adipogenesis of menin-null mESCs is possible and the menin-null adipocytes are larger than normal, suggesting a role for menin in determining fat-cell size.

### 3.2. Gene Expression Profile of mESC-Derived Menin-Null Adipocytes Shows Reduced Expression of Several Differentially Methylated Genes

To examine the consequence of menin loss in lipoma cells, WT and Men1-KO mESC-derived adipocytes were used as a surrogate for gene expression microarray analysis. The top 40 genes differentially expressed in the Men1-KO adipocytes (at least 3-fold change compared with WT adipocytes; *p* < 0.001) were validated by QPCR. Unknown/predicted genes were not considered in this validation set of 40 genes (Table 1). More than 2-fold change was confirmed for 38 genes in Men1-KO versus WT adipocytes (Table 1).

Men1-KO adipocytes showed more than 3-fold downregulation of 12 differentially methylated genes including 3 genes from the* Meg3* locus (*Meg3*,* Mirg*, and* Rian*) and showed more than 6-fold upregulation of 3 genes from the prolactin locus (*Prl2b1*,* Prl7b1*, and* Prl4a1*) (Table 1). Differential expression of genes that regulate the cell cycle has been observed in pancreatic islet *β*-cell tumors of mice with tissue-specific conditional loss of menin [30]. Analysis of gene expression microarray data showed that the expression of cell cycle genes was unaffected in Men1-KO adipocytes (not more than 2-fold change and *p* < 0.001). Also, menin-binding protein partners [13] were not present among the differentially expressed genes.

DNA methylation inversely correlates with a gene activating chromatin modification, the histone H3 lysine-4 trimethyl mark (H3K4me3) [31]. Menin partners with the MLL-protein-complex to place this active mark in chromatin, and loss of menin leads to loss of H3K4me3 on specific genes [18, 32]. Among the 12 genes found to have differential DNA methylation, a comparison with our previously published data [18] showed that only one gene (Meg3) downregulated in mESC-derived menin-null adipocytes also showed significantly reduced histone methylation (H3K4me3) in menin-null mESCs. We have previously shown that menin loss in mESCs resulted in reduced expression of the long noncoding RNA Meg3 by facilitating DNA methylation in the* Meg3* promoter region [33]. These observations suggest a role for menin in regulating H3K4me3 and consequently DNA methylation at specific genes in adipocytes and other MEN1-associated target tissues.

### 3.3. Prolactin Family Genes Are Upregulated in mESC-Derived Menin-Null Adipocytes

**Figure 2:**
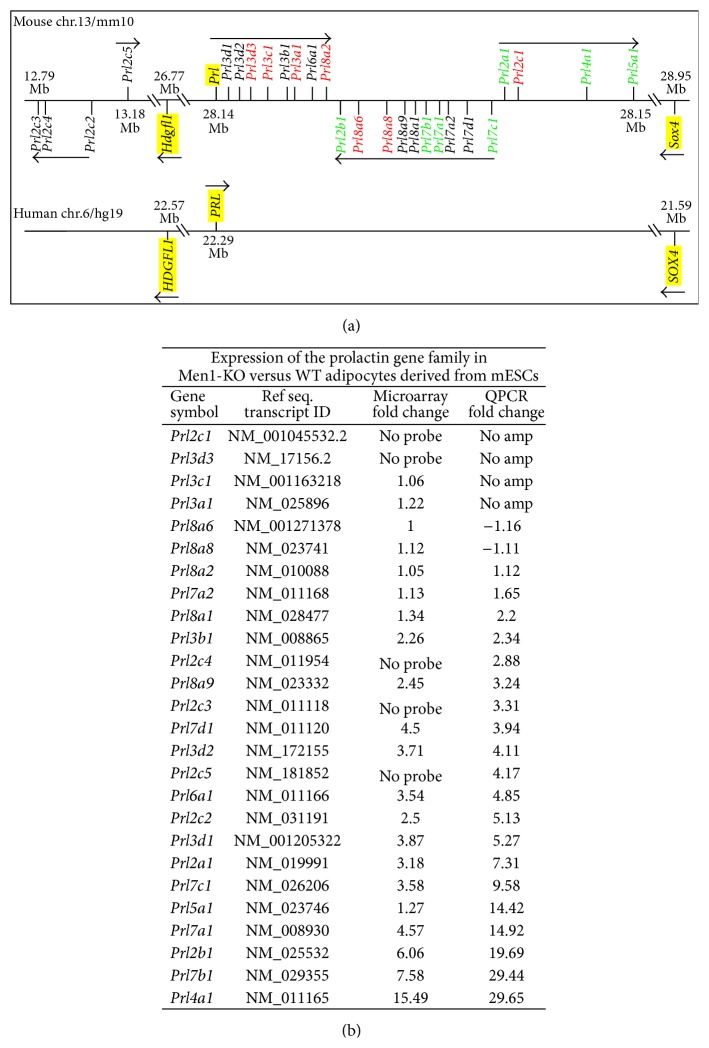
Prolactin family genes are upregulated in adipocytes derived from Men1-KO mESCs. (a) Schematic diagram comparing the mouse prolactin gene family locus with the syntenic human locus. The mouse prolactin gene family locus on chromosome 13 is shown on top and the syntenic human locus on chromosome 6 is shown at the bottom. Red, genes not differentially expressed in Men1-KO versus WT adipocytes or that did not yield a PCR product. Green, genes with the highest fold change (>5-fold) in Men1-KO versus WT adipocytes. Yellow highlight, genes present in both mouse and human. Arrows indicate the orientation of the coding strand. Chromosomal location in megabases (Mb) is from the UCSC genome browser using mouse genome version mm9 and human genome version hg19. (b) Relative expression of prolactin family genes. Prolactin family genes (*n* = 26) were analyzed by QPCR using RNA isolated from adipocytes derived by in vitro differentiation of WT or Men1-KO mESCs. Microarray data and QPCR relative fold change (normalized to Gapdh) are shown. “No probe” indicates genes not present on the microarray, and “no amp” indicates genes that did not yield a PCR product.

The prolactin gene family consists of 26 members clustered in a region of mouse chromosome 13 that includes* Hgdfl1*,* Prl*, and* Sox4* (Figure 2(a)). The syntenic region on human chromosome 6 only contains* HGDF1L*,* PRL*, and* SOX4* (Figure 2(a)). Therefore, the prolactin gene family is unique to the rodent genome. Gene expression microarray analysis showed upregulation of 3 genes from this family in adipocytes derived from Men1-KO mESCs (Table 1). QPCR analysis of all the prolactin family genes (*n* = 26) using RNA isolated from mESC-derived adipocytes showed 1.6- to 29-fold upregulation of 19 genes in menin-null adipocytes; the expression of 3 genes was unchanged, and 4 genes did not yield any PCR product (Figure 2(b)). These data show that loss of menin regulates gene expression at the mouse prolactin gene family locus.

### 3.4. 3T3-L1 Cells with Menin Knockdown Can Undergo In Vitro Differentiation into Adipocytes and Also Show Increased Adipocyte Cell Size

**Figure 3:**
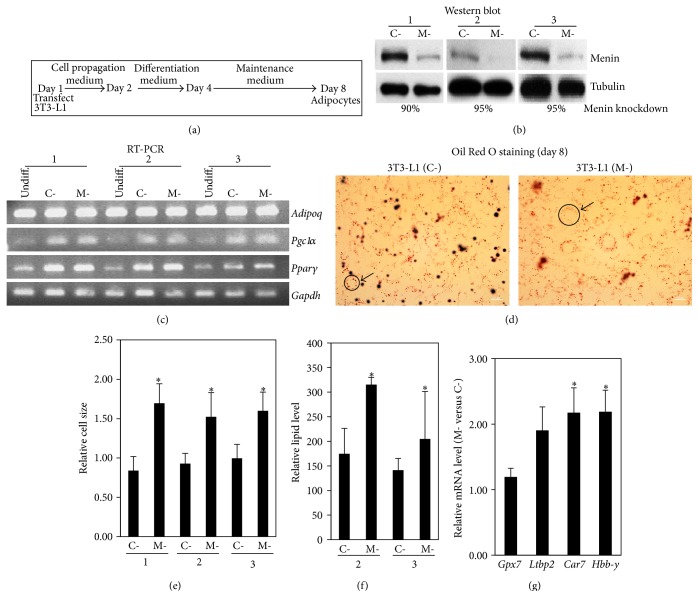
3T3-L1 cells with menin knockdown can also undergo in vitro differentiation into adipocytes and show increased adipocyte cell size. (a) In vitro adipogenesis. Control shRNA plasmid (C-) or Men1 shRNA plasmid (M-) transfected 3T3-L1 cells were plated on day 1 in 3T3-L1 cell propagation medium overnight. The medium was replaced with differentiation medium for 2 days, followed by maintenance medium for 4 days to produce adipocytes, with a medium change (maintenance medium) every two days. (b) Menin knockdown. Whole cell extracts prepared from 3T3-L1 cells differentiated as shown in (a) were analyzed for the level of menin knockdown by western blot (day 8 adipocytes). Antitubulin was used as the loading control. Percent knockdown of menin was calculated after normalization to tubulin. Data are shown for 3 independent experiments. (c) Expression of adipocyte marker genes. RNA isolated from the 3 independent experiments shown in (b) was analyzed for the indicated adipocyte marker genes (*Adiponectin*,* Pgc1α*, and* Pparγ*) and an internal control gene (*Gapdh*) by conventional RT-PCR followed by agarose gel electrophoresis. Lanes marked “undiff” are 3T3-L1 cells before in vitro differentiation. (d) Oil Red O staining. Bright-field microscopy images of C- and M- differentiated 3T3-L1 cells stained for lipids/triglycerides after adipogenesis by Oil Red O staining show round adipocyte cells with lipid droplets (orange colored spots). Images are shown for experiment 2. Magnification = 400x. (e) Relative cell size. Cell diameter was measured as an index of cell size from 3 to 5 microscopy fields using the cell outline formed by the circle of orange lipid droplets accumulated inside the round adipocytes from the 3 independent 3T3-L1 differentiation experiments performed in (b) (an example marked with a black circle and arrow is shown in (d)). Diameter mean and SD are shown (^*∗*^ *p* < 0.05). Note: the units on the *Y*-axis do not reflect the actual diameter of the cells but they reflect the measurement of the cell diameter from images captured at 400x magnification. (f) Relative lipid content. After adipogenesis, Oil Red O staining for lipids/triglycerides was performed, dye was extracted, and the OD was measured at 520 nm. Relative lipid level is shown for 2 independent experiments performed in (b) (OD at 520 nm). Error bar = SD. ^*∗*^ *p* < 0.05. (g) Relative expression of differentially expressed genes identified by microarray analysis of mESC-derived adipocytes. Genes more than 5-fold downregulated (*n* = 8) or upregulated (*n* = 12) in mESC-derived menin-null adipocytes were analyzed by QPCR using RNA isolated from adipocytes derived by in vitro differentiation of 3T3-L1 cells shown in (b) (experiment 2). QPCR relative fold change (normalized to Gapdh) is shown for 4 genes. The other 16 genes did not yield a PCR product in either cell type (C- or M-). ^*∗*^ *p* < 0.05 (>2-fold change in M- versus C-).

To further investigate the results obtained from mESC-derived adipocytes, an independent experimental model of in vitro adipogenesis was employed. The consequence of menin deficiency on adipocytes was studied in 3T3-L1 cells, a well-characterized preadipocyte cell line that is used for in vitro adipogenesis [24] (Figure 3(a)). Menin knockdown in differentiated 3T3-L1 cells was confirmed by western blot (Figure 3(b)). Induction of adipocyte marker gene expression (*Pgc1α* and* Pparγ*) and Oil Red O staining was also observed in adipocytes derived after menin knockdown (Figures 3(c) and 3(d)). Similar to the observation in the Men1-KO mESC-derived adipocytes, the 3T3-L1 adipocytes with menin knockdown showed 1.5- to 1.7-fold increase in cell size with a modest increase in lipid content (Figures 3(e) and 3(f)). Genes with more than 5-fold change in mESC-derived Men1-KO adipocytes (Table 1) were analyzed for expression changes in the 3T3-L1 adipogenesis model. Among the 20 genes analyzed (8 downregulated and 12 upregulated in Men1-KO mESC-derived adipocytes), only 4 were expressed in 3T3-L1-derived adipocytes with or without menin knockdown. Modest expression change (about 2-fold) was observed for 2 of these 4 genes in the menin knockdown 3T3-L1-derived adipocytes (*Car7* and* Hbb-y*) (Figure 3(g)). The expression of 7 prolactin family genes with the highest fold change between mESC-derived Men1-KO and WT adipocytes was also analyzed by QPCR (*Prl4a1*,* Prl7b1*,* Prl2b1*,* Prl7a1*,* Prl5a1*,* Prl7d1*, and* Prl2a1*). Three genes (*Prl4a1*,* Prl5a1*, and* Prl7a1*) gave no product, and no significant differences were observed for the other 4 genes. Therefore, using an independent model of in vitro adipogenesis, we could confirm that menin deficiency did not affect adipocyte differentiation and produced adipocytes with increased cell size. However, the gene expression changes from mESC-derived adipocytes were not observed or could not be investigated in the 3T3-L1 adipogenesis model.

## 4. Discussion

The availability of lipoma specimens from human MEN1 patients or from the mouse models of MEN1 and the availability of normal cells matched to lipoma are a challenge in the study of MEN1-associated lipoma. Here, we demonstrate the potential of using in vitro differentiation to derive menin-deficient and matched normal fat-cells (adipocytes) for such investigations. Using 2 different models of in vitro adipogenesis (mESCs and 3T3-L1 cells), we demonstrate that lack of menin or reduced level of menin did not affect the derivation of adipocytes. Interestingly, we observed that in both models the menin-deficient adipocytes were 1.5- to 1.7-fold larger compared with their WT/menin-containing control adipocytes and with a modest increase in lipid content which perhaps results from the increased cell size and not due to increased production of lipids. Gene expression analysis did not reveal any obviously relevant downstream effectors that could explain fat-cell hypertrophy. Analysis of these cells by posttranscriptional and proteomic approaches will be of interest.

Ppar*γ*-induced in vitro adipogenesis was inefficient in producing adipocytes from immortalized Men1-KO mouse embryo fibroblasts (MEFs) or upon menin knockdown in 3T3-L1 cells [34]. The different methods used for in vitro differentiation in the previous study [34, 35] and in the present study [24, 29] could account for our successful derivation of adipocytes from Men1-KO mESC or from 3T3-L1 with menin knockdown. Similarly, menin inactivation by antisense oligonucleotides did not affect BMP-2-induced commitment of a mouse multipotential mesenchymal stem cell line (10T1/2) and mouse bone marrow stromal cell lines (ST2 and PA6) into the adipocyte lineage [36].

Men1-KO mESC-derived adipocytes showed downregulation of a few differentially methylated genes including the long noncoding RNA Meg3 and upregulation of most of the prolactin family genes. This observation could not be confirmed in the 3T3-L1 adipogenesis model. Meg3 was not expressed in 3T3-L1 or 3T3-L1-derived adipocytes and therefore further decrease from menin knockdown could not be assessed. Similarly, the prolactin family genes analyzed (*n* = 7) were either not expressed in WT or menin-deficient 3T3-L1-derived adipocytes or unaffected upon menin knockdown. The difference in gene expression in mESC or 3T3-L1-derived adipocytes could be attributed to their differentiation ability. Embryonic stem cells are multipotent and able to commit to different cell lineages, and 3T3-L1 cells are already committed to the adipocyte lineage [15, 24]. The reduced expression of several genes that are affected by differential DNA methylation highlights the involvement of menin in the regulation of DNA methylation at specific genes in the mESC-derived adipocytes.

We previously reported that menin loss in mESCs impaired H3K4me3 and gene expression at the* Meg3* locus; and in mESC-derived pancreatic islet-like endocrine cells, menin loss impaired H3K4me3 and gene expression at the four* Hox* loci [18]. Also, in Men1-KO MEFs gene expression and H3K4me3 are significantly reduced at the four* Hox* loci [32]. Consistent with these data, in the present study, we found that, in mESC-derived adipocytes, menin loss increased gene expression from the prolactin gene family locus and decreased the expression of genes from the* Meg3* locus. These observations suggest that the structural organization at loci with gene clusters is a target of menin to up- or downregulate gene expression. Gene rich chromatin domains that contain coordinately expressed genes in clusters such as the* Hox* genes are highly conserved in evolution [19]. However, the prolactin family genes are present in rodents and not in humans [22]. Therefore, the effect from menin loss on the expression of prolactin gene family is not relevant in the context of MEN1-associated human lipoma.

About 30% of MEN1 patients develop lipomas as one of the cutaneous manifestations of the MEN1 syndrome. These patients carry a heterozygous germline mutation in* MEN1*, and loss of the remaining normal copy of* MEN1* has been documented in such MEN1-associated lipomas [5–9]. Our findings from in vitro differentiation studies in mouse cells that adipocytes can form despite menin deficiency are in agreement with the observation that lipomas resemble normal fat. However, how menin loss leads to localized increased fatty mass and tumor formation remains to be determined. Further studies of these benign fatty lesions could provide insights into the pathogenesis of MEN1-related lipomas and the growth of normal fat tissue.

## 5. Conclusion

In vitro adipogenesis showed that menin deficiency could result in fat-cell hypertrophy and differential gene expression from the methylated* Meg3* locus and the prolactin gene family locus. Our data highlight the potential of two different in vitro experimental systems to study MEN1-associated lipoma biology and to investigate the mechanisms that govern fat-cell size, proliferation, and function.

## Supplementary Material

Supplementary Table 1 shows the sequences of all the primers used in this study.

## Figures and Tables

**Table 1:** Differentially regulated genes in Men1-KO versus WT adipocytes derived from mESCs.

Affymetrix probe set ID	Ref seq. transcript ID	Gene symbol	Gene title	Microarray fold change	QPCR fold change
1451634_at	NR_002853	**Airn**	Antisense Igf2r RNA	−9.24	−18.5
1457030_at	NR_028265	**Mirg**	miRNA containing gene	−8.36	−6.54
1438588_at	NM_009538	**Plagl1**	Pleiomorphic adenoma gene-like 1	−7.04	−7.46
1427580_a_at	NR_028261	**Rian**	RNA imprinted and accumulated in nucleus	−6.96	−10.7
1436713_s_at	NR_003633	**Meg3**	Maternally expressed gene 3	−6.72	−7.35
1417836_at	NM_024198	**Gpx7**	Glutathione peroxidase 7	−5.91	−6.49
1421461_at	NM_001122949	Mpl	Myeloproliferative leukemia virus oncogene	−5.68	−1.74
1418061_at	NM_013589	**Ltbp2**	Latent transforming growth factor beta binding protein 2	−5.51	−2.28
1428896_at	NM_026840	Pdgfrl	Platelet-derived growth factor receptor-like	−4.52	−2.9
1419411_at	NM_009312	Tac2	Tachykinin 2	−4.34	−3.29
1423506_a_at	NM_010923	**Nnat**	Neuronatin	−3.92	−2.86
1460412_at	NM_024237	**Fbln7**	Fibulin 7	−3.91	−7.51
1448788_at	NM_010818	Cd200	CD200 antigen	−3.76	−4.5
1421074_at	NM_007825	**Cyp7b1**	Cytochrome P450, family 7, subfamily b, and polypeptide 1	−3.73	−3.09
1419197_x_at	NM_032541	Hamp	Hepcidin antimicrobial peptide	−3.73	−2.84
1417979_at	NM_022322	Tnmd	Tenomodulin	−3.63	−3.36
1425040_at	NM_028593	Cybrd1	Cytochrome b reductase 1	−3.57	−4.69
1419292_at	NM_001042615	**Htra3**	HtrA serine peptidase 3	−3.49	−5.69
1435476_a_at	NM_001077189	**Fcgr2b**	Fc receptor, IgG, low affinity IIb	−3.27	−8.81
1416347_at	NM_001168488	Men1	Multiple endocrine neoplasia 1	−3.18	−714.1
1450285_at	NM_011667	Ube1y1	Ubiquitin-activating enzyme E1, Chr Y 1	4.34	9.38
1435064_a_at	NM_020626	Tmem27	Transmembrane protein 27	4.39	9.44
1448783_at	NM_021291	Slc7a9	Solute carrier family 7 (cationic amino acid transporter, y+ system), member 9	4.43	17.26
1456601_x_at	NM_007503	Fxyd2	FXYD domain-containing ion transport regulator 2	4.51	19.42
1424279_at	NM_001111048	Fga	Fibrinogen alpha chain	4.74	7.31
1418916_a_at	NM_029269	Spp2	Secreted phosphoprotein 2	4.86	19.56
1449907_at	NM_001163028	Bcmo1	Beta-carotene 15,15′-monooxygenase	4.92	2.84
1422289_a_at	NM_029636	Ctsq	Cathepsin Q	4.94	6.32
1418766_s_at	NM_001161355	Timd2	T-cell immunoglobulin and mucin domain-containing 2	5.2	13.92
1443824_s_at	NM_053070	Car7	Carbonic anhydrase 7	5.39	5.89
1417761_at	NM_007468	Apoa4	Apolipoprotein A-IV	5.59	7.67
1418724_at	NM_007686	Cfi	Complement component factor i	5.86	9.12
1440218_at	NM_001033364	Cdhr2	Cadherin-related family member 2	6.02	−1.07
1452165_at	NM_025532	*Prl2b1 *	Prolactin family 2, subfamily b, member 1	6.06	19.69
1451513_x_at	NM_009243	Serpina1a	Serine (or cysteine) peptidase inhibitor, clade A, member 1A	6.28	12.46
1418282_x_at	NM_009244	Serpina1b	Serine (or cysteine) peptidase inhibitor, clade A, member 1B	6.34	6.02
1424673_at	NM_053165	Clec2h	C-type lectin domain family 2, member h	7.19	7.11
1429826_at	NM_029355	*Prl7b1 *	Prolactin family 7, subfamily b, member 1	7.58	29.44
1448572_at	NM_011165	*Prl4a1 *	Prolactin family 4, subfamily a, member 1	15.49	29.65
1436717_x_at	NM_008221.4	Hbb-y	Hemoglobin Y, beta-like embryonic chain	19.31	2.8

Validation of top 20 downregulated or upregulated genes (*p* < 0.001) identified in the gene expression microarray analysis. QPCR fold change was normalized to Gapdh. Bold, genes regulated by differential DNA methylation. Italic, genes from the prolactin gene family.
