# Supplementary material for: Consequence of Menin Deficiency in Mouse Adipocytes Derived by In Vitro Differentiation
Source: Int J Endocrinol. 2015 Jul 2;2015:149826. doi: 10.1155/2015/149826 (PMC4503551; doi:10.1155/2015/149826)
Supplement: Supplementary file 1 — Supplementary Table 1 shows the sequences of all the primers used in this study. [file 149826.f1.pdf]

SUPPLEMENTARY TABLE 1: Primers used for PCR in this study (mouse)		
**Gene**	**Forward primer**	**Reverse primer**
**Microarray validation**		
Airn	CATTATATCTATCCCTCCTTGCTC	CTGCTGATTCATGTTTGCTCTTC
Apoa4	AATCTGCACAGGGACACAGGTA	GTTCTACAGCCTCCTTGGCATT
Bcmo1	ATTTGGGAGGCAGACAGAAGAC	TTTGGCATCCAGAATGAGTAAAAA
Car7	TTCACTGGAACGCCAAGAAGTA	GGAGCCAGGATAGGTCCAGTAG
Cd200	CAAGCATCACCTTCTGGAACAC	CGCAGAGCAAGTGATGTTTAGG
Cdhr2	AGCCCTTTGGACTTTGAGACAG	TGCTGGTGGAGAATTCAGTGTT
Cfi	GATTTCCCAACGAGTCTGTCCTTC	CTTTTGGAATGTTCTGCTGTCG
Clec2h	GCGTAGCATCAAATTCCCATCT	CCTCCTCTCGTGAGAACCAAGT
Ctsq	TGAGACATGACTCCTGCTTTCTTC	TTTCTTCCCATATTGCTCTTCTCA
Cybrd1	AAAAGTCCCCCACATGTACAGC	AAGAGGAGGAGTCCAGAATACACG
Cyp7b1	CACCAGAGAACAATTGGACAGC	CAGTGGAGGAAAGAGGGCTACA
Fbln7	GGCAAGAAGTTTGGAAGCAAGT	CTGGCTGGAGCATTCACTGATA
Fcgr2b	CTCTATCCCAAAAGCCAACCAC	CTTCCTCTGGAAGGGTTTCTCC
Fga	GGAAGGAGAAACTCGCAACACT	AATTCAGCGATCCATCCATTCT
Fxyd2	GGGACAGAGAATCCCTTCGAGT	TTCATCTTCATTGACATGCCTA
Gpx7	ACCAGACACCAACAGGGAGATT	TCCGTCTGGGTCCACTAGGTA
Hamp	GCACCACCTATCTCCATCAACA	CCACACTGGGAATTGTTACAGC
Hbb-y	CTCATCAATGGCCTGTGGAGTA	ACTCTCCAAAAGCAGTCAGCAC
Htra3	GCCGATGTGGTGGAGAAGATTG	GCAACACAGGGAGCTTTTTCTT
Ltbp2	ATTCCAGAGAGTCCAGCAGAAGAG	TACAGTAGTCTTGGCTGGGGTGTA
Meg3	CTTCCTGTGCCATTTGCTGTTG	TGCAACGTGTTGTGCGTGAAG
Men1	CCTCCATCGATCTTCACACTGACT	CTGAGGCAATTCCCTTGTGATA
Mirg	TTTCCTCGATCCTCATGAAACA	GTCAACAGTATGGGGAGGGAAG
Mpl	GATCACCTTGGTGACTGCTCTG	AAGGACTTAGGGCTGCAGTGTC
Nnat	AGGGACACAGCCCATTGCGAGAAG	CACAGGAGCACCTGATGATGACAC
Pdgfrl	AGGGTGTGGTCTACTGCAAAGC	CCTGAATTCAACCTCCACATCA
Plagl1	GCTGTCAAAAATGTGGCAAGTC	GTAGGAGATCTTGTTGGGATCGTG
Prl2b1	TCCTGGTGTCCTACCTGCTTTT	TCTTTCCCTGGGCATACTGATT
Prl4a1	GTACCATCTGTGGGCTGAAATGT	GGCAGTATAGTTCCCGTTTTCG
Prl7b1	GGACACCAGTTTAGCAGCCTTT	CATTTCGCTAACACCTGATCCA
Rian	ACCAGGTTCAAGGTCCCTCATA	TTCCTCACTGTCTTCCATTCCA
Serpina1a	TGGTGGAGAAGTTTCTGGAAGAG	CTTCCTCAGTGTTCTCAGGATCG
Serpina1b	CAGAGGAGGCCAAGAAAGTGAT	CAGTGTTCTCAGGATCGAATGG
Slc7a9	TTCCTCTGGTGACCGTATGCTA	GATGGTTGAAAATGCCACAAAA
Spp2	GTGTACGACTACGACCCTTCCTCT	ATGTGGTTTCCTGAACACTGAACT
Tac2	ACGTGACATGCACGACTTCTTT	AGCAGGCACTGCTTTATG
Timd2	GCTACGGCTCTCTCCTAACTGG	CCACCAAGGTGTGTCGAATAAATA
Tmem27	TCCATGAGAAAAGTTCCCAACA	GCACTGTTGATCCGGTTCCTAT
Tnmd	TCCTGGCCTTAACTCTAATTGTCC	GTCATGGACTTCCAATGTTTCATC
Ube1y1	AGGCTTGTTCTGGAAAGTTTATGC	CTTGCCAAGCTTCTCTTGTAGGTC
**Prolactin gene family**		
Prl2a1	CTGGCAAGTCCCTCTGAACCAT	CAGACCAGCCAGGGTAGTTCTCAT
Prl2b1	TCCTGGTGTCCTACCTGCTTTT	TCTTTCCCTGGGCATACTGATT
Prl2c1	GGAACAAGCCAGGCTCACACAC	TCCGGACTGCGTTGATCTTTTT
Prl2c2	GGAACAAGCCAGGCTCACACAC	TCCGGACTGCGTTGATCTTTTT
Prl2c3	TCCCATGTGTGCAATGAGGAAT	TGCATCTCATGGGGCTTTTGT
Prl2c4	GAACAAGCCAGGCTCACACACT	TCCGGACTGCGTTGATCTTTTT

Prl2c5	GCCCCATGAGATGCCATACTTC	TGTCTGTGGCTTTGGAGATGATT
Prl3a1	CCATGTGCTTTGACATGGAAGG	GCAAGTGAAGTGGTGTGGCAGT
Prl3b1	CCTGGGAAGAGCCTCTGAAACA	ACGCACCGCCATAAGGTTCTAA
Prl3c1	TGGTGTCATGCATGTTCCTGTG	GGTTTGGCACATCTTGGTGTTTT
Prl3d1	CCCCTGTGTCATACTGCTTCCA	TGAAAGACAACTCGGCACCTCA
Prl3d2	TTGGAGCCTACATTGTGGTGGA	ACACATCTGCGGCCAAGATAAA
Prl3d3	TTGGAGCCTACATTGTGGTGGA	ACACATCTGCGGCCAAGATAAA
Prl4a1	GTACCATCTGTGGGCTGAAATGT	GGCAGTATAGTTCCCGTTTTCG
Prl5a1	GAGGTTGCCAGAAGGTCCTCAA	CCATCGAGATCAGCCATGTTGT
Prl6a1	CTAAGGAGGGAGCCCGACAGAT	CTGCAAGGATGCCAGTTCATTC
Prl7a1	TGCCACACTTTGCCACTCAAAT	GGCGCCCCTGGCATACTCTT
Prl7a2	CAGGCTGTGCCAGAACTCTTCA	ATGTCTTGCGCAGTTCCATGTT
Prl7b1	GGACACCAGTTTAGCAGCCTTT	CATTTCGCTAACACCTGATCCA
Prl7c1	CAACAGCTGCCACACATTTTCC	TCCTGATGGCGTTGGCTCTTAT
Prl7d1	AGGCTTTCAACAGCTGCCACAC	ATAAGGGCATTCTGGCAAAGCA
Prl8a1	GTGCCATGAATGATGTCCCTGA	CACACGAAGGCAATGGGAAAGGT
Prl8a2	GGGAGAAAGCTGCATCAATTCCT	GCTCTGAGAACCTCCTCATCACG
Prl8a6	TTTTGTGGGTTCCTGGATCAGC	CCCAGTGGGGAAATTCTTCTGTC
Prl8a8	CCCACGGATGGAAACATTTGAC	AACAATCTCATCCCGCCGAAC
Prl8a9	TGGAACCCTCTCGTGGAAACAT	TGCATGCTCAGTTCCCCTATCA
**Adipogenesis**		
Adipoq	GTTGCAAGCTCTCCTGTTCC	GCTTCTCCAGGCTCTCCTTT
Gapdh	ATCACTGCCACCCAGAAGAC	CAACCTGGTCCTCAGTGTAG
Men1	GTACATGCGCTGCGACCGTAAG	TCATCCTGGTAGTAGGTCTTAG
Pgc1α	CGGTCTTAGCACTCAGAACC	AAGCTCTGAGCAGGGACGTC
Ppparγ	CCCTGGCAAAGCATTTGTAT	AATCCTTGGCCCTCTGAGAT
